# Patients’ views of shared decision making in inflammatory bowel disease: a survey in China

**DOI:** 10.1186/s12911-021-01702-8

**Published:** 2021-12-06

**Authors:** Dingting Xu, Hanyun Zhang, Yan Chen

**Affiliations:** Department of Gastroenterology, The Second Affiliated Hospital, School of Medicine, Zhejiang University, Jiefang Road 88, Hangzhou, Zhejiang 310009 People’s Republic of China

**Keywords:** Shared decision making, Chronic disease, Chinese patient, Online questionnaire, Attitude

## Abstract

**Background:**

Recently, decision-making process has become increasingly complex. But there is limited information on Chinese patients’ views of shared decision making (SDM) in inflammatory bowel disease (IBD). This questionnaire investigation aimed to understand Chinese patients’ perspectives and expectations of SDM in IBD and analyze the possible factors that influence their views.

**Methods:**

An online survey was conducted from July 19th to 24th, 2020. A total of 1118 patients completed the survey.

**Results:**

One-third of patients were dissatisfied with the current decision-making model, and the satisfaction of inpatients was lower than that of outpatients. 84% of patients preferred to participate in SDM, who were young and had a high education level, high income, commercial insurance, strong learning ability and knowledge of SDM. Most of those who did not want to participate (72%) were worried about the cost. The kind of medicine (948, 84.8%), surgical indications (505, 45.2%) and operation methods (482, 43.1%) were the topics that patients thought most require SDM. Side effects of medicine (837, 74.9%), costs of therapy (675, 60.4%), and surgical risks (563, 50.4%) were considered to be the most influential factors for SDM. 52.7% of all patients hoped experts in different disciplines would participate in SDM. The most desirable amount of time for discussion was 30 to 60 min (562/1118, 50.3%), that were associated with the cost of SDM.

**Conclusion:**

We can meet the needs of patients by reducing costs and strengthening online patient education and exploring a model suitable for Chinese IBD patients.

**Supplementary Information:**

The online version contains supplementary material available at 10.1186/s12911-021-01702-8.

## Background

In the last decade, the incidence of inflammatory bowel disease (IBD) in China has increased rapidly [1]. According to data from the Chinese Center for Disease Control and Prevention in 2014, the total number of cases of IBD in China between 2005 and 2014 was about 350,000. That number is still growing. At the same time, the decision-making process for treatment has become increasingly complex. IBD is highly heterogeneous, for example, patients may have different symptoms and complications due to the different site and duration of the disease, each IBD patient needs individualized therapy. Meanwhile, an increasing number of biological agents have been or will be used in the treatment of IBD, including anti-TNF, anti-integrin agents, and anti-interleukin 12/23 antibody [2–4]. In addition, there are also new small-molecule targeted drugs in the clinical validation. These drugs differ in effectiveness and safety. All of these makes the decision more difficult. Shared decision making (SDM) may be a solution.

Shared decision making (SDM) is a process shared between patients and physicians through which patients can become informed about available evidence and weigh the pros and cons of various treatments with medical providers [5]. SDM can meet the needs of patients to participate in-depth in diagnosis and treatment. At the same time, medical providers can involve exploring patients' values and treatment preferences. In addition, SDM can increase adherence and satisfaction and reduce health care costs among patients with IBD [6]. Although the goals of SDM are consistent, there is no standard working mode of SDM. Many scholars have carried out SDM practice of different modes, as well as the development and application of SDM tools [7–9].

On the contrary, the current mode of medical decision making takes the physician as the subject, and patients are treated according to the plan made only by their physicians. But are patients satisfied with this status? As early as 2009, Baars et al. conducted a questionnaire survey with patients in the Netherlands, demonstrating that IBD patients’ desired to be actively involved in the decision-making process [10]. A patient survey was also conducted in Japan in 2016, and the majority of IBD patients preferred SDM according to their perception of the severity or progression of their disease [11]. China, as a newly industrialized country, has many characteristics of an industrialized country, including a medical model, culture, economy, and access to medicine. The demands of patients are also increasing. There are no data on Chinese patients’ views of SDM in IBD. In this survey, we will determine how satisfied patients are with current model. Because of possible differences in decision-making patient received in outpatient and inpatient, satisfaction surveys will be conducted separately. More importantly, our study will aim to understand Chinese patients’ perspectives and expectations of SDM in IBD and analyze the possible factors that influence their views. We expect to provide a novel insight into Chinese IBD patients’ views of SDM.

## Materials and methods

### Participants and recruitment

Patients included in this survey met the following criteria: (1) diagnosis with Crohn’s disease (CD), ulcerative colitis (UC) or indeterminate colitis; (2) informed consent. We recruited patients through *WeChat* patient groups online. The questionnaire was put into 4 *Wechat* groups. When filling out the questionnaire, patients would be asked about the above inclusion criteria. If they failed to meet the criteria, the questionnaire would be automatically terminated.

### Survey development and delivery

The questionnaire was designed by our team (the members are all IBD physicians), based on previous survey studies in Dutch and Japanese IBD patient [10–12], and take into account China's medical situation. SDM-Q-9 [13] was used as reference for the survey of patients' satisfaction with current medical decisions. Then the questionnaire was revised by 3 experienced specialists who feedback their views and modify the questionnaire. After that, we contacted the volunteers from the China Crohn's & Colitis Foundation (CCCF), which is a nonprofit, volunteer-driven organization [14], and invited 3 IBD patients to try out the questionnaire, feedback their opinions and finally finalized the questionnaire. The questionnaire was produced by Wenjuanxing (https://www.wjx.cn), which is a free, open platform for survey design. We delivered the survey online from July 19th to 24th, 2020. The questionnaire content focused on 4 topics, 41 items: (1) patient characteristics (including assessment of disease activity), (2) patient satisfaction with current decision making, (3) patient preferences for SDM, 4) the mode of SDM. All questions were choice tests except age. Some questions, such as whether you prefer to participate in SDM, respondents would be further asked different questions based on their choices (see Additional file 1).

### Assessment of disease activity

The Simple Clinical Colitis Activity Index (SCCAI) was used to evaluate UC activity, and an SCCAI ≥ 5 was considered to indicate active disease [15]. The Harvey-Bradshaw Index (HBI) was used to evaluate CD activity, and an HIB > 4 was considered to indicate active disease [16].

### Statistical analysis

Data were analyzed using IBM SPSS Statistics 26 (IBM SPSS predictive analytic community, USA). Normally distributed data are presented as the mean ± standard deviation (SD), and nonnormally distributed data are presented as the median (minimum–maximum). The χ^2^ test, *t* test, and nonparametric test were used for comparison. Significance was established at *P* < 0.05.

### Ethical considerations

This study was approved by the Medical Ethics Committee of the Second Affiliated Hospital, School of Medicine of Zhejiang University (No. 474).

## Results

### Patient characteristics

A total of 1118 Chinese patients with IBD participated in this online questionnaire survey. The mean (SD) age of patients was 33.8 (11.2) years. A total of 59.5% patients were male. Of these patients, 857 had CD, 213 patients had been diagnosed with UC, and 48 patients had been diagnosed with unclassified colitis. The patient characteristics are shown in Table 1.

**Table 1:** Patient characteristics and preferences

	Overall (percent)	Prefer to participate in SDM	Prefer not to participate	*p* value
Number	1118	945	173	
Percent of all patients		84.5%	15.5%	
Mean age (SD), years	33.8 (11.2)	33.5 (10.8)	35.6 (13.6)	0.021
Male gender	665 (59.5%)	577 (61.1%)	88 (50.9%)	0.012
*Disease type*				0.038
Crohn's disease	857 (76.7%)	735 (77.8%)	122 (70.5%)	
Ulcerative disease	213 (19.1%)	168 (17.8%)	45 (26%)	
Unclassified colitis	48 (4.3%)	42 (4.4%)	6 (3.5%)	
Active CD or UC	245 (26.9%)	210 (25.5%)	35 (23.5%)	0.595
*Duration of disease*				0.083
0–0.5 years	103 (9.2%)	82 (8.7%)	21 (12.1%)	
0.5–5 years	559 (50.0%)	485 (51.3%)	74 (42.8%)	
≥ 5 years	456 (40.8%)	378 (40.0%)	78 (45.1%)	
*Education*				0.000
Elementary school and below	34 (3%)	26 (2.8%)	8 (4.6%)	
Junior high school	194 (17.4%)	144 (15.2%)	50 (28.9%)	
Senior high school and technical secondary school	238 (21.3%)	198 (21.0%)	40 (23.1%)	
Junior college	225 (20.1%)	196 (20.7%)	29 (16.8%)	
Undergraduate	384 (34.3%)	341 (36.1%)	43 (24.9%)	
graduate degree	43 (3.8%)	40 (4.2%)	3 (1.7%)	
*Income*^*a*^				0.008
< 2000 RMB	107 (9.6%)	82 (8.7%)	25 (14.5%)	
2000 RMB–5000 RMB	395 (35.3%)	326 (34.5%)	69 (39.9%)	
5000 RMB–10,000 RMB	356 (31.8%)	308 (32.6%)	48 (27.7%)	
10,000 RMB–20,000 RMB	183 (16.4%)	156 (16.5%)	27 (15.6%)	
> 20,000 RMB	77 (6.9%)	73 (7.7%)	4 (2.3%)	
*Medical insurance*				
Basic medical insurance	804 (71.9%)	690 (73%)	114 (65.9%)	0.055
New rural cooperative medical insurance	341 (30.5%)	273 (28.9%)	68 (39.3%)	0.006
Commercial insurance	57 (5.1%)	54 (5.7%)	3 (1.7%)	0.029
None	11 (1%)	9 (1.0%)	2 (1.2%)	0.803
Has ever been hospitalized (inpatient)	1039 (92.9%)	880 (93.1%)	159 (91.9%)	0.567
Surgical history	400 (35.8%)	338 (35.8%)	62 (35.8%)	0.986
Has not discussed with doctor before surgery	63 (15.8%)	51 (15.1%)	12 (19.4%)	0.251
*Medicine history*				
5-ASA	690 (61.7%)	572 (60.5%)	118 (68.2%)	0.056
Glucocorticoids	388 (34.7%)	329 (34.8%)	59 (34.1%)	0.857
Immunomodulator	618 (55.3%)	519 (54.9%)	99 (57.2%)	0.575
Biological agents	588 (52.6%)	506 (53.5%)	82 (47.4%)	0.137
Nutritional	628 (56.2%)	525 (55.6%)	103 (59.5%)	0.332
Others	120 (10.7%)	102 (10.8%)	18 (10.4%)	0.879
Has joined a self-help group	1107 (99.0%)	936 (99.0%)	171 (98.8%)	0.803
Has never learned IBD knowledge online or through WeChat	59 (5.3%)	43 (4.6%)	16 (9.2%)	0.003
Has never learned IBD knowledge through books	95 (8.5%)	65 (6.9%)	30 (17.3%)	0.000
*Familiarity with SDM*				0.016
Has never heard of it	387 (34.6%)	310 (32.8%)	77 (44.5%)	
Knows about it but not exactly	393 (35.2%)	339 (35.9%)	54 (31.2%)	
Knows the general meaning	292 (26.1%)	252 (26.7%)	40 (23.1%)	
Has a thorough knowledge	18 (1.6%)	18 (1.9%)	0	
Has participated in SDM	28 (2.5%)	26 (2.8%)	2 (1.2%)	

^a^The RMB against the U. S. dollar was 6.99 on July 30th, 2020

### Patient satisfaction with current decision making

We surveyed patients' satisfaction with the current decision-making model. According to the differences between outpatient and inpatient patients in terms of condition and demand, as well as the differences between doctors' working modes, we divided them into groups according to outpatient and inpatient, and asked patients their satisfaction with outpatient and inpatient decision-making modes respectively. The results of Table 2 showed that the satisfaction of inpatients was significantly lower than that of outpatients.

**Table 2:** Patient satisfaction with current decision making: outpatient vs inpatient

	Outpatient (total number, 1118)	Inpatient (total number, 1039)	*p* value
Satisfied with the depth of explanation in decision making	739 (66.1%)	645 (62.1%)	0.001
Satisfied with time invested in current decision making	742 (66.4%)	644 (62.0%)	0.003
Satisfied with the method of current decision making	792 (70.8%)	693 (66.7%)	0.000

### Patient preferences for SDM

Now we need to further understand whether Chinese patients are willing to participate in SDM and what factors influence their choice. The survey showed that the majority of patients (84.5%) reported that they would participate in SDM if there were an opportunity, while 173 patients (15.5%) indicated that they would not participate. We thus divided the population into two groups: “Prefer to participate in SDM” and “Prefer not to participate”. The characteristics of these two groups are shown in Table 1. Patients who preferred to participate in SDM were generally young, or had a high education level, high income, commercial insurance, strong learning ability or more knowledge of SDM. On the other hand, higher percentages of the group of patients who preferred not participate in SDM had new rural cooperative medical insurance and had never heard of SDM.

**Fig. 1:**
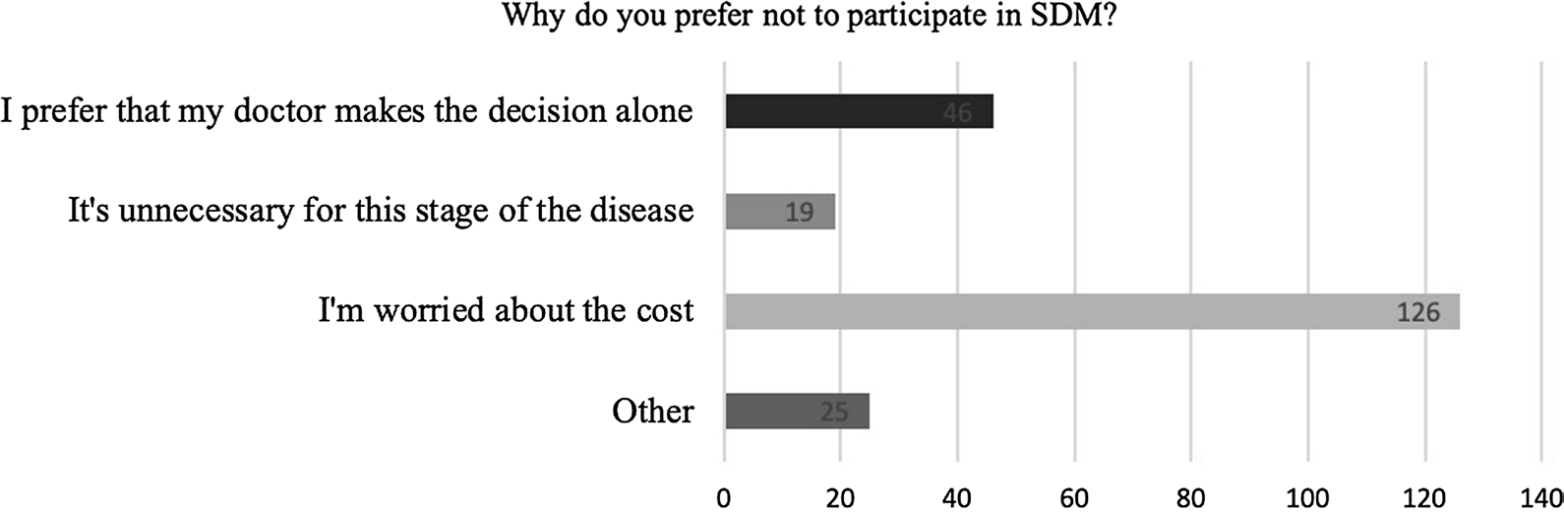
Why do you prefer not to participate in SDM?

Then, we went further and asked the patients who did not want to participate in SDM why they did not want to participate. In this group, 126 patients (72.8%) were worried about the cost (according to the current outpatient consultation cost in China, the consultation cost was about RMB500-800/ hour, which were not covered by insurance), and 46 patients (26.6%) thought the responsibility for decision making about therapy rested only with physicians (Fig. 1).

**Fig. 2:**
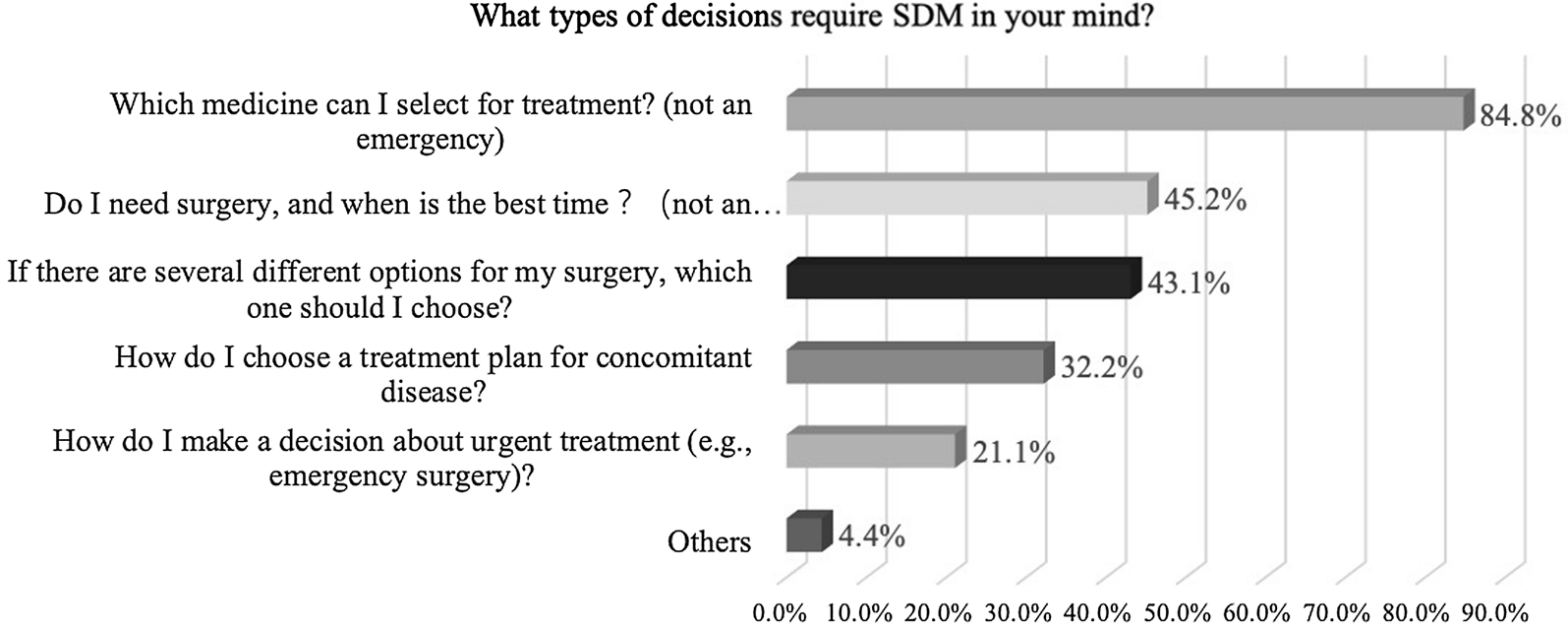
What types of decisions require SDM in your mind?

**Fig. 3:**
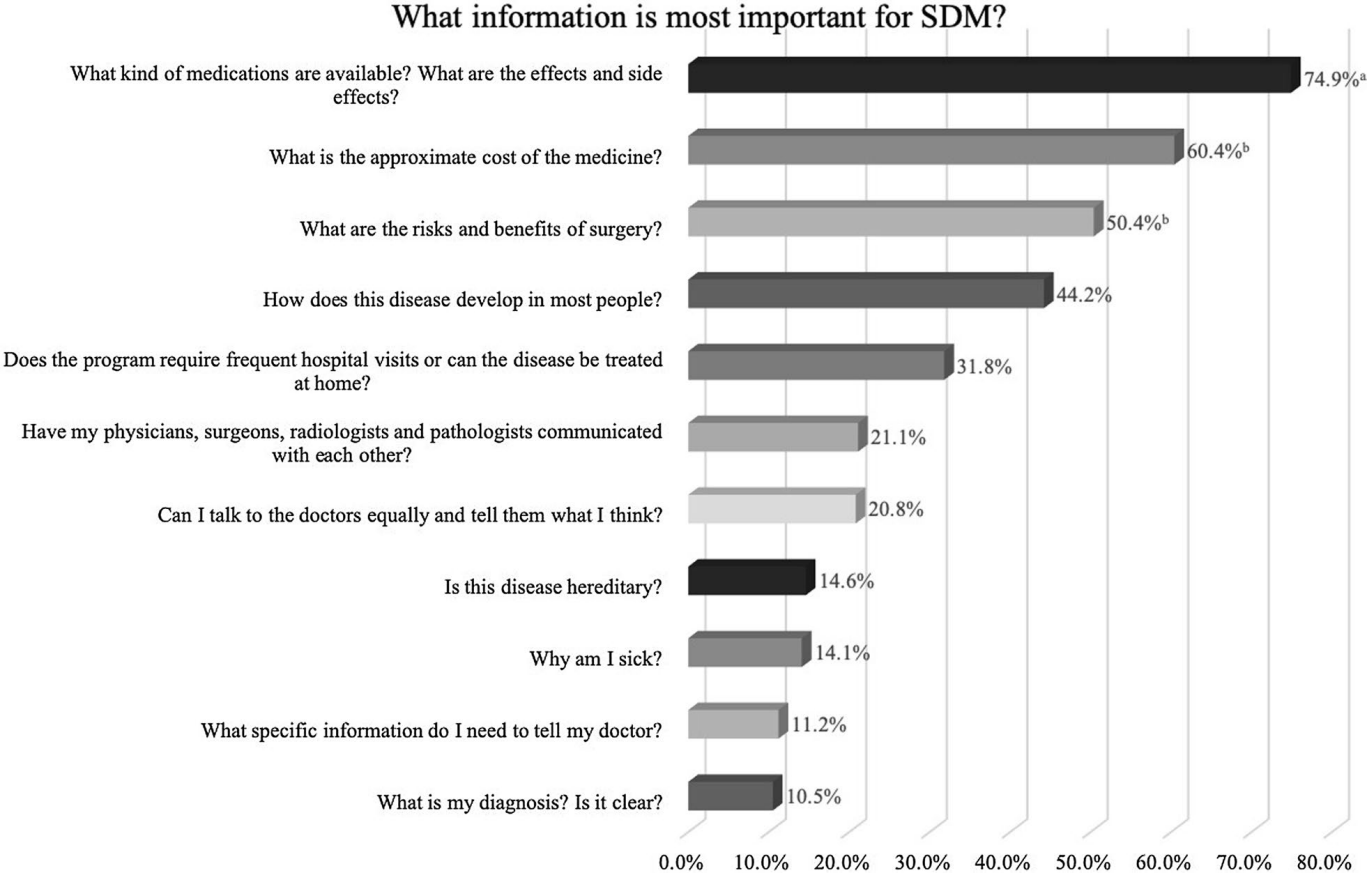
What information is most important for SDM?

We also investigated patients’ opinions about appropriate SDM content, including the types of decisions requiring SDM and information considered important for SDM. All results are shown in Figs. 2 and 3. According to patients, the kind of medicine (948, 84.8%), surgical indications and timing (505, 45.2%) and operation methods (482, 43.1%) were the types of decision that most require SDM. Kind of medicine and effects and side effects of medicine (837, 74.9%), costs of therapy (675, 60.4%), and surgical risks and benefits (563, 50.4%) were considered to be the most influential factors for SDM.

### Mode of SDM

In order to further understand the specific needs of Chinese patients for SDM, such as the desired time, number of participating doctors, issues to be discussed, and affordable costs, etc. We asked patients additional questions on this topic (Table 3). The first question concerned besides physicians, who the patient would like to or was willing to discuss his or her decision making with: 37.7% of 1118 patients wanted to discuss with other patients; 22.8% wanted to discuss with a family member, and 5.3% wanted to make decisions by themselves; 34.8% considered all of the above options to be acceptable. There was difference, but not significant in the responses to this question between patients who were willing or unwilling to participate in SDM. Then, we asked patients how many physicians they would like to be involved in SDM: 52.7% of patients hoped experts in different disciplines, such as physicians, surgeons, radiologists and pathologists, would participate in SDM. In addition, we found that significantly more patients who were unwilling to participate in SDM (38/173, 22%) would prefer to have only one attending physician than patients who were willing to participate in SDM (82/945, 8.7%). The most desirable amount of time for discussion was 30 to 60 min (562/1118, 50.3%). Some patients (39.4%) selected a time of less than 30 min, and others (10.3%) hoped for more than 1 h. Further comparing the patients who preferred and did not prefer to participate in SDM, we found that 56.1% (97/173) of patients who preferred not to participate SDM chose a time of less than 30 min, while 53.4% (505/945) of patients who preferred to participate in SDM chose 30–60 min (*p* < 0.01).

**Table 3:** Mode of SDM

	Overall (percent)	Prefer to participate in SDM	Prefer not to participate	*p* value
Number	1118	945	173	
*Who the patient wanted to or was willing to discuss with*				0.453
Other patients	422 (37.7%)	363 (38.4%)	59 (34.1%)	
A family member	248 (22.8%)	212 (22.4%)	36 (20.8%)	
Only themselves	59 (5.3%)	47 (5.0%)	12 (6.9%)	
All of the above was acceptable	389 (34.8%)	323 (34.2%)	66 (38.2%)	
*How many physicians they would like to be involved in SDM*				0.000
Only one attending physician	120 (10.7%)	82 (8.7%)	38 (22.0%)	
Several experts in gastroenterology	385 (34.4%)	325 (34.4%)	60 (34.7%)	
Several experts in different disciplines	589 (52.7%)	518 (54.8%)	71 (41.0%)	
Others	24 (2.1%)	20 (2.1%)	4 (2.3%)	
*Desirable amount of time for discussion (which was associated with the cost of SDM, if RMB500-800/hour)*				0.000
Less than 30 min	441 (39.4%)	344 (36.4%)	97 (56.1%)	
30 to 60 min	562 (50.3%)	505 (53.4%)	57 (32.9%)	
Longer than 60 min	115 (10.3%)	96 (10.2%)	19 (11.0%)	

## Discussion

An increasing number of patients and physicians believe that SDM is important in IBD [10–12]. There is an increasing number of patients in China who are young and like using the internet, care about information about daily life, disease introduction, drug progress and side effects [17]. As a country with a large population, China is short of medical resources. SDM requires more time and attention from the physicians. We need to understand the views and needs of Chinese patients on SDM before practicing. We designed the questionnaire based on previous studies [10–12, 18] and the characteristics of Chinese patients. The study samples obtained were similar to the previously reported epidemiological characteristics of IBD in China in terms of age and sex ratio after classification by CD and UC [19]. Our survey showed that the majority of patients (84.5%) with IBD would participate in SDM and patients who preferred to participate in SDM were generally young or had a high education level, high income, commercial insurance, strong learning ability or knowledge of SDM. This questionnaire survey was the first to investigate Chinese IBD patients’ preference for SDM.

This study showed more than 90% of the patients who preferred to participate in SDM had learned IBD knowledge online or through books and had knowledge of SDM, which was a larger percentage than that in the group unwilling to participate in SDM in our study. Another survey also showed that more than half of IBD patients used the internet to collect information and that the likelihood of seeking such information was related with age and education level [20]. Patients who are younger and more highly educated are more likely to have access to up-to-date information about IBD and to have difficulty making decisions; in other words, they are in greater need of more and willing to participate in SDM. The results suggest that we can further promote IBD knowledge online but that, at the same time, we should pay attention to people who do not use the internet and provide detailed knowledge when patients visit a health professional. Another recommended approach is *WeChat*, one of the most popular social media sites in China. We have found that *WeChat* has become a major source of information for IBD education in China [17]. Therefore, we could also provide information on SDM based on patients’ demands through *WeChat*. However, it should be noted that our questionnaire was delivered through *Wechat*, which would exclude patients who could not use *Wechat* or had low education level, resulting in biased results. Therefore, it is better to conduct further face-to-face questionnaire survey.

The proportion of patients who preferred SDM in our study was less than that in the Netherlands [10] and Japan [11]. This difference may be related to the study objectives. We examined patients' actual willingness to participate as the research object, while the other two studies took patients' awareness of the importance of SDM as the research object. Among the patients who were less willing to participate, cost was a practical issue for 72.8% of respondents. Some of them may think SDM was important, but chose not to participate because of some factors. Beyond that, 26.6% of patients who preferred not to participate thought the responsibility for decision making about therapy rested only with physicians. These factors may have contributed to the differences between our research and those in other areas.

Our analysis showed a high-income population, and patients with commercial insurance favored SDM. Patients with the new rural cooperative medical insurance were the opposite. In China, there are three insurance schemes implemented by the government [21], including the New Rural Cooperative Medical Scheme (NRCMS) in rural areas and the Urban Employee Basic Medical Insurance (UEBMI) and Urban Resident Basic Medical Insurance (URBMI) in urban areas. These three insurance schemes constitute China's basic social medical insurance system, with universal health insurance coverage successfully achieved by 2011 [22]. Since there is little difference in coverage and reimbursement ratio between UEBMI and URBMI, these two categories are merged into Basic Medical Insurance in our study. Among these schemes, the NRCMS covered more than 830 million farmers by 2011 [22], which had the potential to reduce the burden of medical expenses on individuals and households. However, rural residents covered by the NRCMS have relatively low income and education levels compared with those of people covered by other insurance schemes, and medical expenses have increased [23]. On the other hand, commercial insurance is often used as a supplement to basic insurance, and the income of this part of the population is usually high. These differences may account for the inconsistencies in patients’ intention to participate SDM. However, it should be noted that the number of patients with commercial insurance was small in our study, so the effect of commercial insurance may not be statistically significant. Regarding the reason for a preference not to participate SDM, the cost was reported to be the greatest problem for these patients. The Dutch survey did not involve cost-related issues [10]. Although the Japanese survey showed that there was no correlation between income and whether SDM was important or not [11], it does not show health insurance coverage. An investigation of pediatric physicians indicated that insurance limitations were a barrier [18]. A survey on gastroenterologists’ views of SDM also mentioned the problem of cost, and 19.8% of them believe that lack of payment for services were barriers to SDM implementation [12]. Van Veenendaal et al. suggested changes in the sociopolitical context, including financial services, to accelerate the implementation of SDM in the Netherlands [24]. We believe this is also an issue that needs to be addressed in SDM in China.

At present, SDM is mainly carried out in outpatient departments and may improve outcomes [25, 26]. However, whether inpatients need SDM is unknown. We surveyed patients' satisfaction with current patterns of medical decision making. The results showed that the satisfaction of outpatients was higher than that of inpatients. We believe this finding may be associated with the more complex conditions and higher needs of hospitalized patients. Therefore, SDM can be implemented in hospitalized patients. Blankenburg et al. found that team size, number of learners, patient characteristics, and type of decision being made did not affect inpatient SDM and that any health professional could perform SDM if properly trained [7]. We suggest that it is necessary for the health professional team to spend more time communicating with inpatients. To communicate with patients more quickly and effectively, we could provide patients with relevant disease information from the Internet or hospitals and other information that physicians or patients consider to be important.

Regarding which types of decisions require SDM, patients selected “Which drug can I select for treatment? (not an emergency)” (84.8%) and “Do I need surgery, and when is the best time? (not an emergency)” (45.2%) in this study. In a previous study, gastroenterologists reported that SDM was appropriate in many situations, including “selecting a course of treatment that may have significant risks and benefits” (87%) and “deciding on elective surgical procedures” (78%) [12]. Although studies have reported that provider recommendations do not align with patient preferences [27], the patients and physicians in the study had similar responses to this question. We designed another question about what patients thought was the most important information for SDM. The results revealed that medicine-related information, including the effects, side effects and cost of medicines, were perceived as the most important. It was concluded that medical problems were a major concern for patients and considered to be most important for SDM.

Finally, we surveyed patients about their thoughts on the SDM model, including the amount of time, participants, and consent to consult with other patients. The results showed that patients preferred to involve experts from multiple disciplines. In addition, they thought 30–60 min was appropriate, and most of them were also willing to discuss with other patients. However, notably, the comparison of patients who were willing and unwilling to participate in SDM showed that more patients who were not willing to participate wanted the discussion to be less than 30 min because the discussion time was related to cost. Following the previous discussion, cost may be one of the important factors affecting the attitudes of SDM patients in China, which is also a reminder that this aspect can be improved.

In conclusion, these data offer important information about IBD patients’ preferences and a foundation for the future practice of SDM. Based on this, we can meet the needs of patients by strengthening patient education and promoting the dissemination of disease-related knowledge. Further, we need to inform the public about IBD and promote the support of health insurance policy in order to reduce the financial burden of patients. We hope through these efforts, SDM practices in China will be promoted.

## Supplementary Information

**Additional file 1**. Questionnaire on IBD patients' views of shared decision making (SDM).

## Data Availability

The datasets used and/or analysed during the current study are available from the corresponding author on reasonable request.

## Abbreviations

IBD: Inflammatory bowel disease
SDM: Shared decision making
CD: Crohn’s disease
UC: Ulcerative colitis
