# Supplementary material for: Patients’ views of shared decision making in inflammatory bowel disease: a survey in China
Source: BMC Med Inform Decis Mak. 2021 Dec 6;21:340. doi: 10.1186/s12911-021-01702-8 (PMC8650369; doi:10.1186/s12911-021-01702-8)
Supplement: Supplementary file 1 — Additional file 1. Questionnaire on IBD patients' views of shared decision making (SDM). [file 12911_2021_1702_MOESM1_ESM.docx]

**Title:** Patients’ views of shared decision making in inflammatory bowel disease: A survey in China

**Supplementary Material**

**Questionnaire on IBD patients' views of shared decision making（SDM）**

**Part 1. patient characteristics**

**Your gender: [single choice]**

| ○Male | ○Female |
| --- | --- |

**Your age: [fill in the blank]** _________________________________

**Your highest degree: [single choice]**

| ○Illiterate | ○Elementary school | ○Middle school | ○High school or secondary schoo |
| --- | --- | --- | --- |
| ○Junior college | ○Bachelor degre | ○Master degree or above |  |

**The average monthly household income is about [single choice]**

| ○Less than RMB 2,000 |
| --- |
| ○RMB 2,000 to 5,000 (Excluding 5,000) |
| ○RMB 5,000 to 10,000 (Excluding 10,000) |
| ○RMB 10,000 to 20,000 (excluding 20,000) |
| ○RMB 20,000 or more |

**Do you have medical insurance? (Multiple choice) [multiple choice]**

| □New rural Cooperative Medical Service (Rural Insurance) |
| --- |
| □Basic medical insurance for urban workers (Urban Employee Medical Insurance) |
| □Basic medical insurance for urban residents (Urban Basic Medical Insurance, no jobs) |
| □Medical Insurance for College students |
| □Commercial Insurance |
| □Public health care (retirement) |
| □None |

**Medical expenses mainly come from: [single choice]**

| ○The main source is your own income |
| --- |
| ○The main source is the joint income of yourself and family |
| ○The main source is family income |
| ○Others |

**Do you have any family members with the same disease or similar disease? (Crohn's disease, ulcerative colitis, inflammatory bowel disease?) [single choice]**

| ○No |
| --- |
| ○Yes, there are |

**Are you a volunteer of the China Crohn’s and Colitis Foundation (CCCF)? [single choice]**

| ○yes |
| --- |
| ○no |

**Your disease type is [single choice]**

| ○Ulcerative colitis (No stoma) |
| --- |
| ○Ulcerative colitis (Stoma) |
| ○Crohn’s disease (no stoma) |
| ○Crohn’s disease (Stoma) |
| ○Unclassified inflammatory bowel disease |

**How many times did you use the toilet to defecate on an average day (24 hours) during the previous week? [single choice]**

**Ulcerative colitis, please**

| ○0-3 times/day |
| --- |
| ○4-6 times/day |
| ○7-9 times/day |
| ○more than 9 times/day |

**During the previous week, on average, how many times per night did you get up to use the toilet to defecate? [single choice]**

**Ulcerative colitis, please**

| ○never |
| --- |
| ○1-3 |
| ○more than 3 |

**During the previous week, did you have enough time to go to the bathroom when you needed to? [single choice]**

**Ulcerative colitis, please**

| ○Tolerance |
| --- |
| ○Need to Go to the bathroom quickly |
| ○Go to a nearby restroom immediately |
| ○Fecal incontinence |

**How many times in the previous week did you notice blood in your stool? [single choice]**

**Ulcerative colitis, please**

| ○never |
| --- |
| ○Less than half the time |
| ○Almost half the time |
| ○More than half the time |

**If you choose a number between 1 and 10 to assess your general health status during the previous week, you would choose [fill in the blank]**

**1= very poor，10=perfect，Ulcerative colitis, please**

_________________________________

Any other extra-intestinal complications in the past week? [Multiple choice]

Ulcerative colitis, please

| □Arthritis |
| --- |
| □Uveitis |
| □Erythema nodosum |
| □Pyoderma gangrena |
| □None |

**What do you think of yourself in general? [single choice]**

**Crohn's disease, please**

| ○good |
| --- |
| ○a bit poor |
| ○poor |
| ○bad |
| ○very poor |

**Your abdominal pain: [single choice]**

**Crohn's disease, please**

| ○no |
| --- |
| ○mild |
| ○middle |
| ○server |

**Your present stools: loose stools times a day [fill in the blank]**

**If there is no loose stools or diarrhea, fill in 0. Crohn's disease, please**

**Do you currently have abdominal masses [single choice]**

**Crohn's disease, please**

| ○no |
| --- |
| ○there are suspicious |
| ○Confirm abdominal mass |
| ○Abdominal mass with tenderness |

**Do you currently have the following complications [multiple choice]**

**Crohn's disease, please**

| □Arthritis |
| --- |
| □Iritis |
| □Erythema nodosum |
| □Pyoderma gangrena |
| □Aphthar ulce |
| □Anal fissure |
| □Anal fistula |
| □Anal abscess |
| □None of the above |

**The time you were diagnosed with inflammatory bowel disease (IBD) is approximately [single choice]**

| ○Five years or more |
| --- |
| ○Six months to five years |
| ○1-6 months |
| ○within 1 month |
| ○just diagnosis |

**Which of the following medications have you used or are you using (multiple choice) [multiple choice]**

| □Mesalamine |
| --- |
| □Biological agents |
| □Glucocorticoid (e.g. Methylprednisolone/prednisone/Medrol) |
| □Immunosuppressants (e.g. Azathioprine, methotrexate, thalidomide, cyclosporine, etc.) |
| □Enteral nutrition |
| □others |

**Part 2 patient satisfaction with current decision making**

**Do you know about the diagnosis and treatment of inflammatory bowel disease (IBD) through Internet or wechat? [single choice]**

**Please specify your past or present study frequency**

| ○Search every week |
| --- |
| ○Monthly search |
| ○Search occasionally every year |
| ○Never |

**Do you know about the diagnosis and treatment of inflammatory bowel disease (IBD) through books or brochures? [single choice]**

**Please specify your past or present study frequency**

| ○Study every week |
| --- |
| ○Monthly study |
| ○Study occasionally every year |
| ○Never |

**Have you joined the patient group? Such as wechat group or QQ group [single choice]**

| ○yes，there are |
| --- |
| ○no |

**Do you know "shared decision making（SDM）"? [single choice]**

| ○Haven't heard |
| --- |
| ○I heard about it, but I don t know |
| ○Get the general idea |
| ○In-depth understanding |
| ○Participation in SDM |

**How do you know about SDM？ [single choice]**

| □Online, wechat or Weibo news, etc |
| --- |
| □patients group |
| □Informed by doctors |
| □I saw it before and understood it by myself |

**Is it important to you if your doctor invites you to participate in medical decisions? [single choice]**

**The disease refers to Crohn's disease or ulcerative colitis. Medical decisions include: what treatment to use, what drugs to choose, whether to operate, when to operate, how to operate, etc**

| ○very important |
| --- |
| ○important |
| ○generally |
| ○not important |
| ○Not important at all |

**Are you satisfied with the time your physicians currently spend making medical decisions for you in outpatient?**

**Medical decisions include: what treatment to use, what drugs to choose, whether to operate, when to operate, how to operate, etc**

| ○No satisfaction at all |
| --- |
| ○not satisfied |
| ○the general |
| ○satisfied |
| ○Very satisfied |

**Are you satisfied with the way the physicians present medical decisions for you in outpatient? [single choice]**

**Medical decisions include: what treatment to use, what drugs to choose, whether to operate, when to operate, how to operate, etc**

| ○No satisfaction at all |
| --- |
| ○not satisfied |
| ○the general |
| ○satisfied |
| ○Very satisfied |

**Are you satisfied with the depth of medical decision making provided by the physicians in outpatient? [single choice]**

**Medical decisions include: what treatment to use, what drugs to choose, whether to operate, when to operate, how to operate, etc**

| ○No satisfaction at all |
| --- |
| ○not satisfied |
| ○the general |
| ○satisfied |
| ○Very satisfied |

**Have you ever been hospitalized? [single choice]**

| ○yes |
| --- |
| ○no |

**Are you satisfied with the time your physicians currently spend making medical decisions for you in inpatient?**

**Medical decisions include: what treatment to use, what drugs to choose, whether to operate, when to operate, how to operate, etc**

| ○No satisfaction at all |
| --- |
| ○not satisfied |
| ○the general |
| ○satisfied |
| ○Very satisfied |

**Are you satisfied with the way the physicians present medical decisions for you in inpatient? [single choice]**

**Medical decisions include: what treatment to use, what drugs to choose, whether to operate, when to operate, how to operate, etc**

| ○No satisfaction at all |
| --- |
| ○not satisfied |
| ○the general |
| ○satisfied |
| ○Very satisfied |

**Are you satisfied with the depth of medical decision making provided by the physicians in inpatient? [single choice]**

**Medical decisions include: what treatment to use, what drugs to choose, whether to operate, when to operate, how to operate, etc**

| ○No satisfaction at all |
| --- |
| ○not satisfied |
| ○the general |
| ○satisfied |
| ○Very satisfied |

**How many abdominal surgeries have you had (excluding perianal)? [single choice]**

| ○0 |
| --- |
| ○1 |
| ○2-3 |
| ○4 or more than 4 |

**Has the surgeon ever invited you to discuss specific surgical procedures with him/her during the planning of the procedure [single choice]**

**Anyone who has had surgery, please**

| ○Briefly discussed |
| --- |
| ○Detailed discussion |
| ○No |

**Have you ever been invited to discuss treatment plan other than surgery? [single choice]**

| ○yes |
| --- |
| ○no |

**When doctor is making treatment plans, do you feel it necessary to invite you and your family to discuss together? [single choice]**

**Compared to the doctor telling you what to do**

| ○Absolutely necessary |
| --- |
| ○Necessary |
| ○It doesn't matter |
| ○unnecessarily |
| ○Absolutely unnecessary |

**If you have the opportunity to discuss more deeply with your doctor and develop treatment plans, for example, shared-decision-making clinic, would you attend? [single choice]**

**Shared-decision-making clinic: It can discuss treatment-related content with more doctors at the same time and get more discussion time, but the fee is higher (e.g. RMB 500-800/hour).**

| ○I really want to attend |
| --- |
| ○Somewhat interested |
| ○Do not want to participate |
| ○I don't know |

**The reason you do not want to attend or do not know whether to attend the shared-decision-making clinic, may be [multiple choice] ***

| □The treatment plan should be decided by the doctor |
| --- |
| □My condition is unnecessary |
| □I worry about the high cost |
| □others, e.g. _________________ |

**Part 3. patient preferences for SDM, the mode of SDM**

**Which of the following do you think is most important for shrared-decision-making? [Multiple choice]**

**Please select up to 3 items that you think are most important**

| □What medications do I have? Which one to choose? (Non-emergency) |
| --- |
| □When do I need surgery? When is the best time to operate? (Non-emergency) |
| □If I have several different types of surgery, which one should I choose? |
| □Except Crohn's disease or ulcerative colitis, how to choose the treatment of other co-morbidities? |
| □What are the options for surgical or medical treatment in emergency cases (critical condition)? |
| □others, e.g. _________________ |

**Which of the following information do you think is important in making an individual treatment plan? [Multiple choice] ***

**Please select up to 4 items that you think are most important**

| □How does the disease progress in most people? |
| --- |
| □The risks and benefits of surgery? |
| □What drugs are there? The effects and side effects of the drug? |
| □How much does the drug cost? |
| □Does the program require frequent hospital visits or can it be treated at home? |
| □Are my physicians, surgeons, radiologists, pathologists communicating with each other? |
| □What specific information do I need to give my doctor? |
| □Why do I have this disease? |
| □Is it hereditary? |
| □What is my diagnosis? Is it clear? |
| □Can I communicate equally with my doctor and tell him/her what I think? |

**Which of the following forms of shared-decision-making would you prefer? [single choice]**

| ○I hope to join with other patients for discussion |
| --- |
| ○I want to attend with my family |
| ○participation by myself |
| ○All of these are acceptable |

**How many doctors would be expected to participate in [single choice]**

| ○I only need one of my primary doctors |
| --- |
| ○Several IBD specialists from the department of gastroenterology are preferred |
| ○It is desirable to have IBD specialists in different disciplines, such as surgery, radiology, pathology, nursing, etc |
| ○Other than my primary doctor, I hope _________________ |

**How long would you like to participate in SDM? [single choice]**

***The time will be related to the cost, such as RMB 500-800 per hour***

| ○Within 30 minutes |
| --- |
| ○30-60 mins |
| ○more than 1 hour |

Would you like to share your illness and discuss treatment with other patients with the same disease **in shared-decision-making clinic? [single choice]**

| ○I really want to be with other patients |
| --- |
| ○A little hope |
| ○It doesn't matter |
| ○Don't agree with |

**Which of the following tools would you like to use to aid in shared decision making? [Multiple choice]**

| □Fill out a form to summarize information about the disease before SDM |
| --- |
| □Watch short videos about the disease, such as how to use medication |
| □Provide information on diet, care and other diseases |
| □Can provide the latest international drug related information |
| □There can be software to help SDM |
| □Can provide information related to clinical trials |
| □It doesn't matter |

**Thank you for your answer and for your support!**
